# Is dietary vitamin B intake associated with weight disorders in children and adolescents? The weight disorders survey of the CASPIAN-IV Study

**DOI:** 10.15171/hpp.2019.41

**Published:** 2019-10-24

**Authors:** Roya Taleban, Motahar Heidari-Beni, Mostafa Qorbani, Mohammad Esmaeil Motlagh, Akbar Fazel-Tabar Malekshah, Mohammad Moafi, Neda Hani-Tabaei Zavareh, Roya Kelishadi

**Affiliations:** ^1^Child Growth and Development Research Center, Research Institute for Primordial Prevention of Non-communicable Diseases, Isfahan University of Medical Sciences, Isfahan, Iran; ^2^Non-communicable Diseases Research Center, Alborz University of Medical Sciences, Karaj, Iran; ^3^Department of Pediatrics, Ahvaz Jundishapur University of Medical Sciences, Ahvaz, Iran; ^4^Department of Epidemiology, Provincial Health Center, Mazandaran University of Medical Sciences, Sari, Iran; ^5^Massachusetts College of Pharmacy and Health Sciences, Boston, USA

**Keywords:** Obesity, Overweight, Vitamin B

## Abstract

**Background:** Weight disorders are highly prevalent at the global level. Vitamin B groups are clearly involved in intracellular mechanisms, energy equation, and weight gain. The present study aims to evaluate the association of dietary vitamin B intake and obesity in a large pediatric population.

**Methods:** This cross-sectional study was conducted among children and adolescents, aged 6-18years, living in urban and rural areas of 30 provinces of Iran. The BMI-for-age classifications were as follow: percentile <0.1, (emaciated), 0.1 ≤percentile <2.35 (thin), 2.35 ≤percentile≤84.1 (normal), 84.1 <percentile ≤97.7 (overweight), 97.1 <percentile (obese). A valid 168-item semi-quantitative Food Frequency Questionnaire (FFQ) was used to assess the usual dietary intake including vitamin B.

**Results:** Out of 5606 children and adolescents participated (mean age: 11.62, SD: 3.32),46.8% were girls. The intake of thiamin, pyridoxine, niacin and pantothenic acid increased the likelihood of obesity, compared with the normal-weight group. Odds ratios (ORs) (95% CI) of obesity for vitamin B1, B3, B5, and B6 were 1.32 (1.14-1.53), 1.01 (1.00-1.02), 1.04 (1.00-1.08),and 1.20 (1.04-1.38), respectively. Riboﬂavin, cyanocobalamin, biotin and folic acid did not have any significant association with weight disorders (B2: OR=1.09, 95% CI =0.99-1.20); B12:OR=1.00, 95% CI=0.98-1.03; B8: OR=1.00, 95% CI=0.99-1.00 B9: OR=1.00, 95% CI=1.00-1.00).

**Conclusion:** The current study showed a significant correlation between consumption of vitamin B group and increased risk of excess weight.

## Introduction

Weight disorders are considered as a preventable and highly prevalent disorder while the population of obese and overweight children has been burgeoning in the last four decades.^1^ According to the World Health Organization (WHO), in 2016, about 224 million individuals aged 5-19 were obese or overweight.^2^ A cross-sectional study conducted among 14 400 Iranian children and adolescents aged 7-18 years showed that 9.4% and 11.4% of the participants had overweight and obesity respectively.^3^

Weight disorders associate with lots of health implications and consequent morbidities during the lifetime.^4^ Not only do the obese children tend to experience weight disorders in their adulthood ages, but also they are more likely to be affected by chronic diseases, including diabetes mellitus and cardiovascular diseases.^4-6^ Childhood obesity potentially causes low self-esteem and poor educational attainment.^4,5,7,8^

Children’s weight disorder is generally attributed to a positive balance of energy equation, which is strongly affected by the high intake of energy-dense foods. The balance of energy equation is also influenced by food micronutrients.^9-12^ Micronutrients include vitamins and minerals, consumed at tiny levels. These nutritional elements are involved in various intracellular reactions, regulating psychological status and food intake as well.^11-13^ Different studies showed that excessive amounts of micronutrient intake have been affecting a large proportion of the human population worldwide.^14^ High levels of micronutrient intakes have been caused by various factors, including (i) availability of the out-of-season vegetables and fruits, (ii) more consumption of meat-centric diets, and (iii) more ingestion of vitamin enriched drinks and crops.^14^

The association of the vitamin B intake and obesity has been evaluated in some of the previous studies.^14-17^ For example, it was shown that long-term consumption of vitamin B had a positive association with high prevalence of obesity in the US citizens.^17^ Similarly, the introduction of vitamin B-fortified food has led to a sharp increase in obesity in China.^14^ On the contrary, thiamin and folate deficiencies are frequently reported in obese individuals.^15,16^ A study, conducted in Norway, showed that morbidly obese patients had lower concentrations of pyridoxine, compared with normal subjects.^15^ To the best of our knowledge, none of the previous studies have evaluated the association of vitamin B intake and obesity amongst Iranian adolescents and children. The current study aimed to evaluate the association of dietary intake of thiamin (B_1_), riboflavin (B_2_), niacin (B_3_), pantothenic acid (B_5_), pyridoxine (B_6_), biotin (B_8_), folic acid (B_9_) and cyanocobalamin (B_12_) with obesity and overweight in a nationally representative sample of children and adolescents.

## Materials and Methods

The cross-sectional study resulted as a part of a national project, termed Childhood and Adolescence Surveillance and Prevention of Adult Non-communicable Disease IV (CASPIAN IV). The study was conducted from 2011-2012 and the related protocols were thoroughly described elsewhere.^18^ Briefly, this school-based study was conducted among students that were recruited through clustering sampling method from urban and rural areas of 30 provinces of Iran. In each province 187 participants were recruited (17 clusters of 11 participants), resulting in 5610 participants in the national levels.

The study aims and protocols were clearly described for the participants and their guardians. Afterward, verbal and oral consent was obtained from students and their parents respectively. The study was implemented under the supervision of professional healthcare providers.

Usual dietary intakes were collected using a validated 168-item semi-quantitative food frequency questionnaire (FFQ). In fact, the participants were questioned about their frequencies of food intakes and nutritional consumption over the last year. FFQ included a list of different foods, with a standardized proportion, to inquire about the frequency of consumption for every food.

In addition, weight, and height of the participants were determined with calibrated equipment and standardized protocols. The weight and height were measured to the nearest 0.1 kg and 0.1 cm respectively. Waist circumference was measured through an un-stretchable tape put at the mid-point between the last rib and upper part of the iliac crest. This measurement was taken to the nearest 0.1 cm.

Body mass index (BMI) was calculated by quotient found by dividing the weight by height squared. WHO growth chart was used for BMI-for-age classifications.^19^ The BMI-for-age classifications were as follow: percentile <0.1 (emaciated), 0.1 ≤percentile <2.35 (thin), 2.35 ≤percentile ≤84.1 (normal), 84.1 <percentile ≤97.7 (overweight), 97.1 <percentile (obese).^19^

Abdominal obesity was calculated through the waist-to-height ratio. The participant was considered to have abdominal obesity if the corresponding waist-to-height ratio was more than 0.5.^20^ The amount of each food item was converted into gram using a household scale guide and calculated for one day. We used NUTRITION 4 software (First Databank, San Bruno, CA) for determining the amount of vitamin intake. The US Food and Nutrition Board protocol was used to determine the percentage of the males and females who did not fulfill the recommended of daily allowance (RDA) values of vitamin B. In this study, those who received any supplement were excluded.^21^

### Statistical analysis

All of the data were analyzed using IBM SPSS Statistics for Windows version 20 (IBM SPSS Statistics, Armonk, USA). Regarding the results of the Kolmogorov–Smirnov test, showing non-normal distribution, vitamin B intakes of five different groups were compared through the Kruskal–Wallis test. The association of vitamin B intake and BMI, considered as an independent variable, was evaluated through multinomial logistic regression while the confounding variables were adjusted in two models. In the first model, age and calorie intake were adjusted. In the second model, age, calorie intake, educational levels, physical activity, and family size were controlled. Accordingly, the odds ratios (ORs) and their 95% confidence intervals (CIs) were reported. *P* value of less than 0.05 was assumed as significant.

## Results

Out of 5606 children adolescents participated, 46.8% were girls (Table 1). The percentages of the participants who did not fulfill RDA are presented in Table 2. In the boys, the mean intakes of vitamin B_1_, B_2_, B_3_, B_6_, and B_12_ significantly differed between the five subgroups of BMI (p values for vitamin B_1_, B_2_, B_3_, B_6_, and B_12_ were 0.001, 0.007, 0.020, 0.008, and 0.007 respectively) (Table 3).

Compared with the normal-weight group, thiamine intake increased the odds (95% CI) of obesity and overweight 1.11 (1.00-1.22) and 1.32 (1.14-1.53) times respectively. The similar results were found when the confounding variables were controlled. By gender stratification, in boys, thiamin intake raised the odds of overweight and obesity, compared with those having normal weight (OR, 95% CI: 1.19 [1.03-1.37] and 1.34 [1.10-1.64] respectively). However, in girls, only the odds of obesity were significant (OR = 1.31, 95% CI = 1.05-1.64) (Table 4).

The gender-stratified model showed that riboflavin and cyanocobalamin increased the odds of overweight in boys, compared with the normal-weight group (B_2_: OR = 1.17, 95% CI = 1.06-1.29); B_12_: OR = 1.02, 95% CI = 1.00-1.04) (Table 4).

Niacin and pantothenic acid increased the odds of obesity, compared with the normal-weight group. The similar results were found in the two models when the confounder factors were controlled (B_3_: OR = 1.01, 95% CI = 1.00-1.02; B_5_: OR = 1.04, 95% CI = 1.00-1.08). After gender stratification, our results showed that, in boys, niacin and pantothenic acid raised the odds of obesity, compared with those having normal weight (B_3_: OR = 1.02, 95% CI = 1.00-1.04; B_5_: OR = 1.06, 95% CI = 1.01-1.12) (Table 4).

Compared with the normal-weight group, pyridoxine intake increased the odds (95% CI) of obesity and overweight 1.10 (1.01-1.19) and 1.20 (1.04-1.38) respectively. Gender stratification analysis showed that, in boys, pyridoxine intake raised the odds of obesity and overweight, compared with those having normal weight (OR = 1.25, 95% CI = 1.03-1.52 and 1.18, 95% CI = 1.34-1.33, respectively) (Table 4).

Compared with those who did not have abdominal obesity, thiamin intake increased the odds (95% CI) of abdominal obesity 1.15 (1.04-1.27) times. By gender stratification, it was shown that thiamin intake increased the odds of abdominal obesity in boys (OR = 1.22, 95% CI = 1.07-1.39) (Table 4).

By the gender stratification, B2 and B6 consumption were significantly associated with waist-to-height ratio in boys (B_2_: OR = 1.09, 95% CI = 1.00-1.19; B_6_: OR = 1.14, 95% CI = 1.02-1.28) (Table 5).

## Discussion

The present study shows an association between vitamin B groups and weight disorders in children and adolescents. According to our findings, vitamin B_1_, B_3_, B_5_, and B_6_ intake potentially contribute to weight disorders. In an animal study, it was shown that higher amounts of thiamin intake raised mice obesity, increasing food consumption.^22^ Likewise, the positive association between thiamin intake and weight gain was shown in a case report study.^23^ The association of thiamin intake on weight gain could be attributable to a biochemical food-intake regulator, called AMP-activated protein kinase (AMPK). AMPK, a serine/threonine protein kinase produced in the hypothalamus, enhances food intake and body weight. In this regard, animal studies showed that higher levels of thiamin intake up-regulated AMPK production, leading to further weight gain.^22^

Under normal physiological conditions, weight disorders could be also affected by energy expenditures.^22,24^ Energy expenditure is mainly regulated by mitochondrial membrane protein-called uncoupling protein (UCP1) or thermogenin, existed in brown adipose tissue.^24^ Animal studies showed that higher levels of thiamin intake not only reduce expression of UCP1 but also results in fewer amounts of energy expenditure and further weight gain. In this context, it was plausible that we found a positive association between thiamin intake and obesity.^24^

However, some of the previous studies showed that thiamin might have an inverse association with obesity.^24,25^ For instance, a cross-sectional study demonstrated that obese individuals had significantly lower levels of plasmatic thiamin, compared with normal ones.^25^ The discrepancy found between our results and previous studies might be attributable to sample size as well as the demographic variables (age and sex).

The results of our study showed that boys, receiving further amounts of riboflavin and cyanocobalamin, had higher BMIs. Our findings were in accordance with some of the previous studies.^26-28^ For example, a cohort study conducted among the US population showed that long-term intake of B vitamins might lead to the increased prevalence of obesity.^26^ Likewise, a prospective study, included 235 880 participants from 10 European countries, showed that riboflavin had a positive association with excess weight.^28^ The positive association found between riboflavin and obesity might be attributed to decreased apoptosis of adipocytes. In this context, it was shown that higher levels of riboflavin induced increased lipogenesis and lipid accumulation as well as decrease free fatty acid release.^26,28^

Our findings showed that the intakes of niacin and pantothenic acid were positively associated with weight disorders. Likewise, the positive association between vitamin B_3_ and obesity was found among American children.^26^ Interestingly, world obesity map shows that the US and Canadian citizens - who have received niacin-fortification regimens - are more likely to have obesity, compared with Norwegians, who have not been fed on niacin-fortification diets.^14^ The role of niacin in weight gain might be attributed to glucose-controlling hormones, including insulin, and glucagon as well as glucagon-like peptides.^14,26^ It seems that the excessive amounts of niacin intake might decrease serum glucose concentration, potentially affecting food intake behavior.^14,26^ Besides, it is well established that niacin could be considered as an appetite stimulator, contributing to the higher prevalence of obesity. It has been shown that elderly people, suffering from niacin deficiency, may lose their taste and smell perception, which potentially lead to poor appetite.^14,31,29-31^

Our results indicated a positive association between pyridoxine intake and excess weight. It is well documented that pyridoxine, as a cofactor, is involved in biosynthesis reactions, transforming proteins to fat.^14,32^ That is why pyridoxine intake could enhance obesity in human and animal models.^14,32,33^

Biotin is clearly involved in the fatty acid and amino acid methabolisms.^34^ Some research showed that biotin deprivation inhibits acetyl CoA carboxylase, impeding long fatty acid synthesis.^34,35^ As a result, low levels of biotin intake could inhibit lipogenesis.^36^ On the other hand, an animal experiment indicated an inverse association between biotin intake and weight of adipose tissue, induced by low expression of lipogenic genes.^37^ In the current study, we did not find any association between biotin intake and adiposity. However, further research is warranted to determine the role of biotin in excess weight.

In this research, we did not find any association between folic acid intake and weight disorders. Prospective evaluation of folic acid supplementation showed that folate usage did not induce any significant difference in weight loss.^38^ However, most of the studies showed that folic acid had an inverse association with excess weight.^39-41^ It seems that the ethnicity and age of the participants can deeply affect the positive role of folate in obesity management. Given the fact that weight disorders are deeply affected by different genetic and environmental factors, comprising ethnicity, age, and lifestyle of the participants, further studies are warranted.

### Study limitations and strengths

This was a cross-sectional study, not proving the causal relationships between vitamin B intake and obesity. Using a self-reported FFQ could be considered as a subject of recall bias; however, we used a complete and valid FFQ in a large population to increase the quality of the findings. The other limitation was that we could not examine the serum levels of vitamin B and other biomarkers. On the other hand, the novelty in the Middle East and North Africa region as well as the provision of data on a large nationally-representative sample could be considered as the study strength.

## Conclusion

Overall, the present study supports the notion that higher levels of vitamin B group induce excess weight.

## Ethical approval

The study was approved by the Isfahan University of Medical Sciences Ethical Committee on human experimentation (Project code# 194049) and was conducted in accordance with the Helsinki Declaration of 1964, as revised in Brazil 2013.

## Competing interests

The authors declare that they have no competing interests.

## Funding

This study was funded by Isfahan University of Medical Sciences, as part of a national school-based surveillance program.

## Authors’ contributions

All authors have contributed in designing and conducting the study. RT, MM, MH, MQ, ME, AF, RK, and NH collected the data and RK, RT, and MM did the analysis. All authors have assisted in preparation of the first draft of the manuscript or revising it critically for important intellectual content. All authors have read and approved the content of the manuscript and are accountable for all aspects of the work.

## Acknowledgments

We forward our sincere thanks to the students, parents, and school staff, contributed to the implementation of this national.

**Table 1:** Characteristics of study participants

	**Total**	**Boys**	**Girls**	***P*** **value**
Age (y)	11.61 (3.233)	11.53 (3.32)	11.70 (3.12)	0.04
Weight (kg)	43.09 (17.20)	43.22 (18.12)	42.94 ± 16.09	0.54
BMI (kg/m^2^)	19.03 (4.62)	18.75 (4.45)	19.34 (4.80)	<0.001
Waist circumference (cm)	67.20 (11.52)	67.89 (12.15)	66.42 (10.71)	<0.001
Energy (kcal/day)	2554.25 (781.70)	2520.88 (796.38)	2524.85 (769.95)	0.72

**Table 2:** Percentages of the participants who did not fulfill RDA

	**Name of the vitamin**	**Percentages of participants who did not meet RDA**
B1	Boy	0.5
	Girls	2.8
B2	Boys	7.2
	Girls	4.2
B3	Boys	10.19
	Girls	7.08
B5	Boys	1.1
	Girls	27.5
B6	Boys	14.4
	Girls	8.4
B8	Boys	28.3
	Girls	27.4
B9	Boys	8.59
	Girls	8.26
B12	Boys	6.3
	Girls	5.7

Abbreviation: RDA, recommended daily allowance.

**Table 3:** Comparison of mean intake of B vitamins among BMI categories in Iranian children and adolescents

**B Vitamins intake**		**Emaciated(n = 90)**	**Thin** **(n = 275)**	**Normal weight** **(n = 3591)**	**Overweight** **(n = 894)**	**Obese(n = 525)**	***P*** **value** ^a^
B_1_ (mg/d)	Total	2.05 ( 1.05)	2.04 ( 1.11)	2.23 ( 1.27)	2.38 ( 1.44)	2.28 ( 1.27)	<0.001
	Boys	1.99 ( 0.94)	2.07 ( 1.14)	2.27 ( 1.36)	2.51 ( 1.66)	2.26 ( 1.04)	0.003
	Girls	2.12 ( 1.20)	2.01 ( 1.08)	2.19 ( 1.17)	2.25 ( 1.18)	2.31 ( 1.53)	0.159
B_2_ (mg/d)	Total	2.45 ( 1.27)	2.50 ( 1.45)	2.69 ( 1.57)	2.86 ( 1.72)	2.73 ( 1.49)	0.007
	Boys	2.36 ( 1.23)	2.51 ( 1.45)	2.68 ( 1.57)	3.02 ( 1.86)	2.67 ( 1.39)	0.002
	Girls	2.57 ( 1.32)	2.48 ( 1.46)	2.70 ( 1.56)	2.71 ( 1.55)	2.79 ( 1.63)	0.437
B_3_ (mg/d)	Total	21.27 ( 9.80)	21.17 ( 10.83)	23.04 ( 12.16)	23.90 ( 13.59)	23.75 ( 12.29)	0.020
	Boys	21.36 ( 9.31)	20.71 ( 9.99)	23.04 ( 12.35)	24.28 ( 13.99)	23.93 ( 11.36)	0.044
	Girls	21.45 ( 10.55)	21.69 ( 11.75)	23.04 ( 11.94)	23.52 ( 13.18)	23.52 ( 12.42)	0.478
B_5_ (mg/d)	Total	5.72 ( 2.38)	5.65 ( 2.47)	5.65 ( 2.33)	5.69 ( 2.38)	5.91 ( 2.30)	0.151
	Boys	5.60 ( 2.39)	5.72 ( 2.40)	5.63 ( 2.45)	5.65 ( 2.44)	5.99 ( 2.35)	0.176
	Girls	5.89 ( 2.39)	5.56 ( 2.55)	5.68 ( 2.31)	5.72 ( 2.31)	5.82 ( 2.25)	0.679
B_6_ (mg/d)	Total	2.18 ( 1.35)	2.21 ( 1.28)	2.36 ( 1.44)	2.35 ( 1.63)	2.39 ( 1.34)	0.008
	Boys	2.12 ( 1.22)	2.22 ( 1.36)	2.34 ( 1.46)	2.62 ( 1.78)	2.32 ( 1.22)	0.015
	Girls	2.26 ( 1.52)	2.20 ( 1.18)	2.37 ( 1.42)	2.44 ( 1.47)	2.47 ( 1.49)	0.462
B_8_ (mcg/d)	Total	32.43 ( 16.66)	32.73 ( 17.09)	34.15 ( 17.49)	35.48 ( 17.90)	34.54±16.69	0.085
	Boys	31.12 ( 16.69)	32.87 ( 16.78)	34.29 ( 17.94)	36.19 ( 18.67)	34.21 ( 16.48)	0.082
	Girls	34.24 ( 16.67)	32.58 ( 17.52)	33.99 ( 16.96)	34.77 ( 17.09)	34.97 ( 17.01)	0.691
B_9_ (mcg/d)	Total	580.13 ( 274.56)	569.25 ( 295.45)	587.44 ( 278.31)	596.99 ( 302.55)	600.30 ( 268.39)	0.160
	Boys	564.43 ( 251.60)	562.52 ( 274.89)	588.95 ( 283.49)	598.46 ( 303.23)	621.44 ( 255.97)	0.060
	Girls	601.62 ( 305.37)	577.09 ( 318.66)	585.73 ( 272.40)	595.54 ( 282.24)	572.11 ( 282.24)	0.577
B_12_ (mcg/d)	Total	5.49 ( 3.16)	6.09 ( 5.06)	6.43 ( 4.98)	6.88 ( 5.33)	6.46 ( 4.68)	0.007
	Boys	5.35 ( 3.05)	6.42 ( 5.30)	6.51 ( 5.32)	7.34 ( 5.95)	6.25 ( 3.76)	0.007
	Girls	5.68 ( 3.33)	5.71 ( 4.75)	6.34 ( 4.56)	6.42 ( 4.60)	6.75 ( 5.68)	0.117

Note: Values are mean (SD). ^a^Comparison of the mean intakes of B vitamins among BMI categories using Kruskal-Wallis test.

**Table 4:** Correlation between intakes of vitamin B and BMI categories in Iranian children and adolescents

**Type of B Vitamin**		**Emaciated**	**Thin**	**Normal Weight**	**Overweight**	**Obese**
**B**_1_						
Model 1	Total	0.83 ( 0.62-1.12)	0.89 ( 0.72-1.09)	1	1.11 ( 1.00-1.22)^*^	1.32 ( 1.14-1.53)^***^
	Boys	0.86 ( 0.51-1.43)	0.88 ( 0.66-1.17)	1	1.19 ( 1.03-1.37)^*^	1.34 ( 1.10-1.64)^**^
	Girls	0.87 ( 0.59-1.27)	0.87 ( 0.65-1.18)	1	1.02 ( 0.89-1.18)	1.31 ( 1.04-1.64)^**^
Model 2	Total	0.83 ( 0.61-1.11)	0.88 ( 0.72-1.08)	1	1.11 ( 1.00-1.22)^*^	1.32 ( 1.14-1.53)^***^
	Boys	0.85 ( 0.51-1.43)	0.88 ( 0.66-1.16)	1	1.19 ( 1.03-1.37)^**^	1.34 ( 1.10-1.64)^**^
	Girls	0.85 ( 0.58-1.25)	0.87 ( 0.65-1.16)	1	1.02 ( 0.89-1.18)	1.31 ( 1.05-1.64)^**^
B_2_						
Model 1	Total	0.86 ( 0.70-1.06)	0.95 ( 0.83-1.10)	1	1.06 ( 0.99-1.14)	1.10 ( 0.99-1.21)
	Boys	0.89 ( 0.64-1.25)	1.00 ( 0.82-1.22)	1	1.17 ( 1.06-1.29)^**^	1.12 ( 0.98-1.2)
	Girls	0.88 ( 0.67-1.15)	0.91 ( 0.74-1.10)	1	0.97 ( 0.88-1.06)	1.07 ( 0.93-1.23)
Model 2	Total	0.85 ( 0.70-1.05)	0.95 ( 0.83-1.09)	1	1.06 ( 0.99-1.13)	1.09 ( 0.99-1.20)
	Boys	0.89 ( 0.63-1.25)	1.00 ( 0.82-1.22)	1	1.17 ( 1.06-1.29)^**^	1.12 ( 0.97-1.28)
	Girls	0.86 ( 0.65-1.13)	0.90 ( 0.74-1.10)	1	0.96 ( 0.87-1.06)	1.07 ( 0.93-1.22)
**B**_3_						
Model 1	Total	0.98 ( 0.96-1.01)	0.99 ( 0.97-1.00)	1	1.00 ( 0.99-1.01)	1.01 ( 1.00-1.02)^*^
	Boys	0.99 ( 0.96-1.03)	0.98 ( 0.96-1.00)		1.00 ( 0.99-1.01)	1.02 ( 1.00-1.04)^*^
	Girls	0.98 ( 0.95-1.01)	0.99 ( 0.97-1.01)	1	1.00 ( 0.99-1.01)	1.00 ( 0.99-1.01)
Model 2	Total	0.98 ( 0.96-1.01)	0.99 ( 0.97-1.00)	1	1.00 ( 0.99-1.01)	1.013 ( 1.00-1.02)^*^
	Boys	0.99 ( 0.96-1.03)	0.98 ( 0.96-1.00)	1	1.00 ( 0.99-1.01)	1.02 ( 1.00-1.04)^*^
	Girls	0.98 ( 0.95-1.01)	0.99 ( 0.97-1.01)	1	1.00 ( 0.99-1.01)	1.00 ( 0.99-1.01)
**B**_5_						
Model 1	Total	1.01 ( 0.92-1.10)	0.99 ( 0.94-1.05)	1	1.01 ( 0.97-1.04)	1.04 ( 1.00-1.08)^*^
	Boys	1.00 ( 0.88-1.13)	1.02 ( 0.95-1.10)	1	1.00 ( 0.96-1.05)	1.06 ( 1.01-1.12)^*^
	Girls	1.03 ( 0.90-1.18)	0.96 ( 0.89-1.05)	1	1.01 ( 0.96-1.05)	1.02 ( 0.96-1.08)
Model 2	Total	1.00 ( 0.92-1.10)	0.99 ( 0.94-1.05)	1	1.01 ( 0.97-1.04)	1.04 ( 1.00-1.08)^*^
	Boys	1.00 ( 0.88-1.13)	1.02 ( 0.95-1.10)	1	1.00 ( 0.96-1.05)	1.06 ( 1.01-1.12)^**^
	Girls	1.02 ( 0.89-1.17)	0.96 ( 0.89-1.04)	1	1.00 ( 0.96-1.05)	1.02 ( 0.96-1.08)
**B**_6_						
Model 1	Total	0.90 ( 0.69-1.18)	1.06 ( 0.86-1.30)	1	1.10 ( 1.01-1.19)^*^	1.20 ( 1.04-1.38)^**^
	Boys	1.12 ( 0.67-1.85)	1.13 ( 0.85-1.50)	1	1.18 ( 1.04-1.33)^**^	1.25 ( 1.03-1.52)^*^
	Girls	0.88 ( 0.65 -1.20)	0.99 ( 0.75-1.32)	1	1.02 ( 0.90-1.14)	1.17 ( 0.95-1.43)
Model 2	Total	0.90 ( 0.69-1.17)	1.06 ( 0.86-1.30)	1	1.10 ( 1.01-1.19)^*^	1.20 ( 1.04-1.38)^**^
	Boys	1.12 ( 0.67-1.87)	1.13 ( 0.85-1.50)	1	1.18 ( 1.04-1.33)^**^	1.25 ( 1.03-1.52)^*^
	Girls	0.87 ( 0.64-1.18)	0.99 ( 0.75-1.30)	1	1.02 ( 0.90-1.14)	1.16 ( 0.95-1.42)
**B**_8_						
Model 1	Total	0.99 ( 0.98-1.01)	0.99 ( 0.99-1.00)	1	1.00 ( 0.99-1.00)	1.00 ( 0.99-1.00)
	Boys	0.99 ( 0.97-1.01)	0.99 ( 0.98-1.01)	1	1.00 ( 0.99-1.01)	1.00 ( 0.99-1.01)
	Girls	1.00 ( 0.97-1.02)	0.99 ( 0.98-1.01)	1	1.00 ( 0.99-1.00)	1.00 ( 0.99-1.01)
Model 2	Total	0.99 ( 0.98-1.00)	0.99 ( 0.99-1.00)	1	1.00 ( 0.99-1.00)	1.00 ( 0.99-1.00)
	Boys	0.99 ( 0.97-1.01)	1.00 ( 0.98-1.01)	1	1.00 ( 0.99-1.01)	1.00 ( 0.99-1.01)
	Girls	0.99 ( 0.97-1.01)	0.99 ( 0.98-1.01)	1	1.00 ( 0.99-1.00)	1.00 ( 0.99-1.01)
**B**_9_						
Model 1	Total	1.00 ( 0.99-1.00)	1.00 ( 0.99-1.00)	1	1.00 ( 1.00-1.00)	1.00 ( 1.00-1.00)
	Boys	1.00 ( 0.99-1.00)	1.00 ( 0.99-1.00)	1	1.00 ( 1.00-1.00)	1.00 ( 1.00-1.00)
	Girls	1.00 ( 0.999-1.00)	1.000 ( 0.99-1.00)	1	1.000 ( 1.00-1.00)	1.00 ( 0.99-1.00)
Model 2	Total	1.00 ( 0.99-1.00)	1.00 ( 0.99-1.00)	1	1.00 ( 1.00-1.00)	1.00 ( 1.00-1.00)
	Boys	1.00 ( 0.99-1.00)	1.00 ( 0.99-1.00)	1	1.00 ( 1.00-1.00)	1.001 ( 1.00-1.00)
	Girls	1.00 ( 0.99-1.00)	1.00 ( 0.99-1.00)	1	1.00 ( 1.00-1.00)	1.00 ( 0.99-1.00)
**B**_12_						
Model 1	Total	0.94 ( 0.88-1.00)	1.00 ( 0.97-1.03)	1	1.01 ( 0.99-1.02)	1.00 ( 0.98-1.03)
	Boys	0.94 ( 0.86-1.04)	1.01 ( 0.98-1.05)	1	1.02 ( 1.00-1.04)^*^	1.00 ( 0.97-1.03)
	Girls	0.94 ( 0.85-1.04)	0.97 ( 0.91-1.03)	1	0.99 ( 0.97-1.02)	1.02 ( 0.98-1.05)
Model 2	Total	0.94 ( 0.88-1.01)	1.00 ( 0.97-1.03)	1	1.01 ( 0.99-1.02)	1.00 ( 0.98-1.03)
	Boys	0.95 ( 0.86-1.04)	1.01 ( 0.981-1.058)	1	1.02 ( 1.00-1.04)^*^	1.00 ( 0.97-1.03)
	Girls	0.93 ( 0.85-1.03)	0.972 ( 0.91-1.03)	1	0.998 ( 0.97-1.02)	1.02 ( 0.98-1.05)

Note: Values are OR (95% CI). Model 1: Analyzed by multinomial logistic regression adjusted for age and energy intake (kcal) as covariates. Model 2: Analyzed by multinomial logistic regression adjusted for age, energy intake (kcal), education, physical activity, and family size as covariates. (*P < 0.05, **P < 0.01, ***P<0.001).

**Table 5:** Correlation between intakes of vitamin B and obesity according to waist to height ratio in Iranian children and adolescents

**Type of B vitamin**	**Model 1**			**Model 2**		
	**Total**	**Boy**	**Girl**	**Total**	**Boy**	**Girl**
B_1_	1.15 (1.04-1.27)^**^	1.22 (1.06-1.39)^**^	1.07 (0.93-1.22)	1.15 (1.04-1.27)^**^	1.22 (1.07-1.39)^**^	1.07 (0.93-1.23)
B_2_	1.03 (0.97-1.09)	1.09 (0.99-1.19)	0.97 (0.89-1.06)	1.03 (0.99-1.10)	1.09 (1.00-1.19)^*^	0.97 (0.88-1.06)
B_3_	1.00 (0.99-1.00)	1.00 (0.99-1.01)	1.00 (0.99-1.01)	1.00 (0.99-1.00)	1.00 (0.99-1.01)	1.00 (0.99-1.01)
B_5_	1.00 (0.97-1.03)	0.99 (0.95-1.03)	1.02 (0.97-1.06)	1.00 (0.97-1.03)	0.99 (0.95-1.03)	1.02 (0.97-1.06)
B_6_	1.07 (0.99-1.16)	1.14 (1.01-1.28)^*^	1.01 (0.90-1.12)	1.07 (0.99-1.16)	1.14 (1.02-1.28)^*^	1.01 (0.90-1.12)
B_8_	1.001 (0.99-1.00)	1.00 (0.99-1.00)	1.00 (0.99-1.00)	1.00 (0.99-1.00)	1.00 (0.99-1.00)	1.00 (0.99-1.00)
B_9_	1.00 (1.00-1.00)	1.00 (1.00-1.00)	1.00 (0.99-1.00)	1.00 (1.00-1.00)	1.00 (1.00-1.00)	1.00 (0.99-1.00)
B_12_	1.00 (0.98-1.01)	1.00 (0.98-1.02)	1.00 (0.97-1.02)	1.00 (0.98-1.01)	1.00 (0.98-1.02)	1.00 (0.97-1.02)

Note: Values are OR (95% CI). Model 1: Analyzed by multinomial logistic regression adjusted for age and energy intake (kcal) as covariates. Model 2: Analyzed by multinomial logistic regression adjusted for age, energy intake (kcal), education, physical activity, and family size as covariates. (*P < 0.05, **P < 0.01, ***P<0.001).
